# Complete genome sequence of a new *Mycobacterium* sp. nov. from a compromised Japanese individual

**DOI:** 10.1128/mra.00421-25

**Published:** 2025-12-29

**Authors:** Nobuhiro Asai, Yuriko Igarashi, Narimi Miyazaki, Asami Osugi, Yuki Matsumoto, Shota Nakamura, Satoshi Mitarai, Hiroshige Mikamo

**Affiliations:** 1 Department of Clinical Infectious Diseases, Aichi Medical University 12703, Nagakute, Aichi, Japan; 2 Department of Infectious Control Diseases, Aichi Medical University 12703, Nagakute, Aichi, Japan; 3 The Research Institute of Tuberculosis, Japan Anti-Tuberculosis Association, Tokyo, Japan; 4 Department of Infection Metagenomics, Bioinformatics Center, Research Institute for Microbial Diseases, Osaka University 13013, Suita, Japan; 5 Department of Basic Mycobacteriology, Graduate School of Biomedical Sciences, Nagasaki University 12961, Nagasaki, Japan; University of California Riverside, Riverside, California, USA

**Keywords:** *Mycobacterium nagakutense*, nagakute

## Abstract

Here, we report the complete genome sequencing of a new *Mycobacterium* sp. nov. from a compromised Japanese individual with hematological malignancy. This will represent the essential data for future phylogenetic and comparative genome studies and will be useful for better understanding the evolution of the pathogen.

## ANNOUNCEMENT

We report the complete genome sequence of *Mycobacterium* sp. strain AMU20-3851, which was first clinically isolated from a human with hematological malignancy. This genome sequence report was approved by the Ethics Committee, Aichi Medical University School of Medicine (AMU 2025-182). The clinical isolate was obtained at our institute, located in Nagakute City, Aichi Prefecture, Japan. The pathogen was isolated after 90 h in blood culture (BD BACTEC FX blood culture system, Becton Dickinson, Sparks, MD, USA). The strain was grown with Ogawa growth medium at 37°C. DNA was purified with the standard phenol-chloroform method after enzymatic (100 µg/mL proteinase K with 2% SDS) treatment by bead beating (0.2 mm diameter zirconia beads) (1). The genome sequence was performed by the Kazusa DNA Research Institute (Kisarazu, Japan) on commission of PacBio Sequel II (Pacific Biosciences, USA). For PacBio Sequel II sequencing, DNA was fragmented using g-TUBE (Covaris, USA), sequencing libraries were prepared by using the PacBio SMRTbell express template prep kit 2.0 (Pacific Biosciences), and libraries between 8 and 20 kb were selected using BluePippin (Sage Science, USA). Circular Consensus Sequencing (CCS) was performed with a Sequel II (Pacific Biosciences) using the PacBio Sequel II sequencing kit v2.0 (Pacific Biosciences). High-fidelity (HiFi) reads were generated from the obtained subreads using the CCS package v6.4.0 in PacBio tools distributed via bioconda (2). Sequence quality was assessed using longQC (3).

A total of 3,267,053 subreads were obtained, from which 201,863 HiFi reads were generated. The HiFi reads were assembled into one circular contig by using the hifiasm 0.16.1-r375 (4) and rotated using the circulator v1.5.5 (5) to start at *dnaA,* whose sequence was predicted by prokka v1.14.6 (6, 7). The genome was annotated using the NCBI Prokaryotic Genome Annotation Pipeline. Default software parameters were used unless otherwise noted. Average nucleotide identity (ANI) analysis was calculated using FastANI v1.34 (8, 9).

The chromosome of AMU20-3851 is 6,056,820 bp in length with 68% G + C content, encoding 5,822 predicted genes, and sequenced to an average depth of 311×. The ANI for this strain was 90.5% for *Mycobacterium diernhoferi* and 84.4% for *Mycobacterium frederiksbergense*, the raw data and the assembled genome were deposited in NCBI GenBank and SRA databases (the accession no. GCF_043974665.1). This genome sequence will represent the essential data for future phylogenetic and comparative genome studies and will be useful for better understanding the evolution of the new species, *Mycobacterium* sp. strain AMU20-3851.

## Data Availability

This complete genome sequence and sequence raw data have been deposited at GenBank under the accession no. GCF_043974665.1 and raw PacBio reads in the SRA database under SRR28129243.
